# In *Arabidopsis thaliana* Cadmium Impact on the Growth of Primary Root by Altering SCR Expression and Auxin-Cytokinin Cross-Talk

**DOI:** 10.3389/fpls.2017.01323

**Published:** 2017-07-27

**Authors:** Leonardo Bruno, Marianna Pacenza, Ivano Forgione, Liam R. Lamerton, Maria Greco, Adriana Chiappetta, Maria B. Bitonti

**Affiliations:** ^1^Dipartimento di Biologia, Ecologia e Scienze della Terra, Università della Calabria Arcavacata di Rende, Italy; ^2^School of Biosciences, University of Cardiff Cardiff, United Kingdom

**Keywords:** cadmium, root growth, stem cell niche, auxin/cytokinin cross-talk, SCR expression

## Abstract

Cadmium is one of the most widespread pollutant in both terrestrial and marine environment, and its inhibitory effect on plant growth has been largely demonstrated. However, the molecular mechanisms underlying Cd toxicity in plant and mainly in root, as the first organ sensing soil heavy metals, need to be better investigated. To this aim, in the present work we analyzed the growth and the organization of *Arabidopsis thaliana* primary root in seedlings exposed to Cd (25 and 50 μM) for 8 days starting from germination. Root length, root meristem size, and organization were evaluated together with the behavior of some of the major molecular players in root growth and patterning. In particular, by using different GFP transgenic lines, we monitored: (i) the expression pattern of WOX5 and SCR transcription factors involved in the establishment and maintenance of stem cell niche and in the control of meristem size; (ii) the expression pattern of the IAA-inducible *pDR5::GFP* reporter, PIN 1, 2, 3, 7 auxin carriers and *TCSn::GFP* cytokinin-sensitive sensor as relevant components of hormone circuit controlling root growth. We report that Cd exposure inhibits primary root growth via affecting RAM stem cell niche and root radial pattern. At the molecular level, an impairment of auxin maximum accumulation at the root tip, related to a down-regulation and mislocalisation of PIN proteins, and an enhancement of *TCSn::GFP* cytokinin-sensitive sensor signal is also detected under Cd treatment, thus suggesting an alteration in the homeostasis of auxin/cytokinin signaling. Moreover, and for the first time Cd toxicity on root growth and pattern has been related to a misexpression of SCR transcription factors which is known to interplay with auxin/cytokinin cross-talk in the control of RAM maintenance and activity.

## Introduction

In the modern world, human activities such as extensive mining, industry and improper agricultural practices have led to a release of a great amount of heavy metals to the environment. Cd, in particular, is one of the most widespread heavy metals in both terrestrial and marine environments, and represents an extremely significant pollutant due to its high toxicity and large solubility in water (Gallego et al., 2012; Song et al., 2017).

In both plants and animals, Cd absorption induces complex changes at genetic, biochemical and physiological levels, which account for its toxicity (Jin et al., 2003; Herbette et al., 2006; Liu et al., 2011; Greco et al., 2012). In plants, the most obvious effect of Cd toxicity is a reduction of plant growth related to an inhibition of photosynthesis, respiration and nitrogen metabolism, as well as to a reduction in water and nutrient uptake (dos Santos et al., 2012). At the molecular level, the most recent findings evidenced that many signaling molecules and plant growth regulators are involved in cadmium sensing and downstream plant response, which encompasses modifications at both transcriptional and post-translation levels (Sanità Di Toppi et al., 2003; Sofo et al., 2013; Chmielowska-Bąk et al., 2014; Yue et al., 2016). Epigenetic mechanisms have been also found to be related to the response of plant to Cd (Greco et al., 2012). However, the relationship between all these factors remains somehow unclear, differing in relation to species, plant organ, heavy metal concentration and treatment duration (reviewed by, Chmielowska-Bąk et al., 2014). Therefore, further insight is required mainly with respect to the cross-talk between different hormone classes.

In the present work, we focused the attention on plant root system which, as the first organ sensing soil heavy metals, is strongly affected by Cd. In particular, Cd heavily inhibits primary root growth, while lateral root formation is somehow stimulated (Xu et al., 2010; Yuan and Huang, 2016). There are evidences that the inhibitory effect on primary root is linked to an impairment of auxin optimal accumulation at the root tip which in turn reduces the size and activity of root apical meristem (RAM) (Blilou et al., 2005; Hu et al., 2013; Yuan and Huang, 2016). An increased level of auxin in the whole root has been instead related to a reduced growth and higher root branching under Cd treatment (Sofo et al., 2013). On the other hand, auxin accumulation in the RAM relies on the metabolism and mainly on the transport of the hormone which is achieved over both long distance, through the vascular system, and at short distance, which involves a cell-to-cell mechanism and it is mostly polar (Grieneisen et al., 2007; Petrásek and Friml, 2009). This exclusive polar transport is mediated by an array of membrane transporters including efflux carriers PIN-FORMED (PIN), influx carriers AUX1/LAX and ATP-binding cassette subfamily B (Petrásek and Friml, 2009; Yang and Murphy, 2009). In *Arabidopsis thaliana*, the PIN family consists of eight members which are divided into two subclasses in relation to the length of a hydrophilic loop in the middle of their polypeptide chain. Canonical “long” PINs (PIN1–4 and 7) show mostly polar localization on plasma membrane (PM), and direct auxin transport function is strongly supported for PIN1, 2, 3, 4, and 7 (Müller et al., 1998; Friml et al., 2002a,b; Blilou et al., 2005; Petrásek and Friml, 2009). The polar localization of these proteins on plasma membrane determines the direction of auxin flow (Wiśniewska et al., 2006) playing a relevant role in many auxin-dependent physiological and developmental processes (Luschnig et al., 1998; Blilou et al., 2005; Scarpella et al., 2006; Wiśniewska et al., 2006). According to the relevant role of PIN proteins in auxin transport (Blilou et al., 2005; Grieneisen et al., 2007; Petrásek and Friml, 2009), recently the Cd-dependent alteration of auxin signaling in the root has been related to their mislocalization (Yuan and Huang, 2016; Yue et al., 2016).

On the other hand, plant growth and development depends on the interplay between different phytohormones, and the *in vivo* importance of auxin and cytokinin antagonistic interaction in root patterning and organogenesis has been deeply elucidated using *A. thaliana* model plant (reviewed by, Werner and Schmülling, 2009; Pacifici et al., 2015; Chaiwanon et al., 2016; Slovak et al., 2016). More precisely, it has been demonstrated that cytokinins promote cell differentiation at the transition zone (TZ) suppressing auxin transport, while auxin promotes cell division, suppressing cytokinin signaling (Dello Ioio et al., 2007, 2008; Ruzicka et al., 2009; Perilli and Sabatini, 2010; Perilli et al., 2010). This interplay relies on the opposite control exerted by the two hormone classes on the same target gene, *SHORT HYPOCOTYL* 2 (*SHY2*), which negatively regulates *PIN 1,3,7* genes (Benjamins and Scheres, 2008; Dello Ioio et al., 2008). In particular, auxin drives SHY2 protein degradation through an ubiquitin-ligase complex (SCF^TIR1^), thus stabilizing PIN expression level and therefore auxin export. Conversely, cytokinins promote *SHY2* expression, through the cytokinin receptor ARABIDOPSIS HISTIDINE KINASE3 (AHK3) and the downstream signaling components ARABIDOPSIS RESPONSE REGULATOR1 (ARR1) and ARR12. Interestingly, in the framework of hormone circuits involved in root growth regulation, it is becoming even more clear that also other hormones, such as brassinosteroids (BR) and gibberellins (GA), control root growth by modulating auxin level and distribution (reviewed by, Pacifici et al., 2015; Chaiwanon et al., 2016; Slovak et al., 2016).

In this context, the present work was addressed to further clarify the effects induced by Cd on the genetic network and hormone circuits involved in root growth. In particular, we aimed to investigate: (i) whether Cd-induced reduction of RAM size was related to an impact on the stem cell niche (SCN), whose maintenance is essential for root growth and pattern (Stahl and Rüdiger, 2010); (ii) whether and how such alterations were related to changes in the spatial pattern of auxin/cytokinin signaling. Therefore, by using different GFP transgenic lines which allow to define the histological domains of gene expression, we analyzed: (i) the expression pattern of WOX5 and SCR as markers of quiescent center (QC) and QC/endodermis specification, respectively; (ii) the expression pattern of the IAA-inducible *pDR5::GFP* reporter, PIN 1, 2, 3, 7 auxin carriers and cytokinin-sensitive two-component *TCSn::GFP* sensor (Zürcher et al., 2013).

So far, Cd-depending effects on plant root have been especially investigated on seedlings germinated on water and then exposed to Cd (Besson-Bard et al., 2009; Sofo et al., 2013; Chmielowska-Bąk et al., 2014; Yuan and Huang, 2016). Taking into account that in open field the plants continuously face with Cd presence starting from seed germination, we planned to investigate the above mentioned aspects in roots of *A. thaliana* seedlings germinated in presence of this heavy metal and exposed to a prolonged treatment.

## Materials and Methods

### Plant Materials and Growth Conditions

Seeds of *A. thaliana* (L.) Heynh. ecotype Columbia (Col) and of the transgenic lines *pSCR::SCR-GFP* (Sabatini et al., 1999); *pDR5::GFP* (Ottenschläger et al., 2003), *TCSn::GFP* (Zürcher et al., 2013), *pWOX5::GFP* (Blilou et al., 2005), *pPIN1::PIN1-GFP* (Benková et al., 2003), *pPIN2::PIN2-GFP* (Blilou et al., 2005), *pPIN3::PIN3-GFP* (Blilou et al., 2005), *pPIN7::PIN7-GFP* (Blilou et al., 2005) were used.

Seeds were surface sterilized and grown as reported in Bruno et al. (2015). Briefly, the seeds were incubated in absolute ethanol for 2 min and 1.75% hypochlorite solution (NaClO) for 12 min. After thorough washing with sterile distilled water (3 min × 5 min), the seeds were sown on Petri dishes containing germination medium (GS), 1% sucrose (Valvekens et al., 1988) and 0.7% plant cell culture agar (Sigma-Aldrich). The plated seeds were left at 4°C for 48 h to ensure uniform germination, and then incubated vertically in a growth chamber at 21°C, under 16 h light (150 μmol m^-2^ s^-1^) and 8 h dark and 60% relative humidity. For Cd treatment, CdCl_2_ was dissolved in sterile water and 100 mM stocks were prepared. An aliquot of this stock solution was added directly to the germination medium immediately before placing it in the Petri dishes to prepare the desired Cd concentrations (25 and 50 μM).

### Root Length and Meristem Size Analysis

*Arabidopsis thaliana* seedlings germinated on control (Ctrl) and on Cd containing medium (25–50 μM respectively) and grown in a vertical position were used. Three independent replicates were performed and for each sample, a minimum of 70 seedlings was analyzed. Data were statistically evaluated by Student’s *t*-test.

Root length was monitored until 21 days after germination (DAG) by scanning the plates and analyzing the resulting images through the open source processing program ImageJ^1^.

For the meristem size analysis, seedlings at 8 DAG were stained with propidium iodide following the MPS-PI-staining protocol (Truernit et al., 2008). Confocal images of median longitudinal sections were obtained using a Leica inverted TCS SP8 confocal scanning laser microscope, with a 40x oil immersion objective, and excitation and emission wavelength were 600 and 640 nm, respectively. For meristem size evaluation, the distance and the number of cortex cells in a file extending from the QC to the first elongated cortex cell were measured (Dello Ioio et al., 2007; Perilli and Sabatini, 2010). Three independent replicates were performed, and for each sample a minimum of 70 seedlings was analyzed. Data were statistically evaluated by using one-way ANOVA with Tukey *post hoc* test (*P* ≤ 0.05) after Shapiro–Wilk normality test.

### Confocal Visualization of GFP Expression

Green fluorescent protein expression was monitored in seedlings of the above transgenic lines of *A. thaliana* germinated on control (Ctrl) and on Cd containing medium (25–50 μM respectively) and sampled at 8 DAG. Confocal images of median longitudinal sections were obtained using a Leica inverted TCS SP8 confocal scanning laser microscope, with a 40x oil immersion objective. The detection of Green Fluorescence Protein (excitation peak centered at about 488 nm, emission peak wavelength of 509 nm) was performed by combining the settings indicated in the sequential scanning facility of the microscope. Three independent replicates were performed and a minimum of 40 seedlings was analyzed for each sample.

### Quantification of GFP Signal

Measurements of GFP signal intensity were carried out on the root apex of transgenic lines *pDR5::GFP* and *TCSn::GFP*, in seedlings grown in Ctrl conditions and under Cd 50 μM treatment from germination to 8 DAG. Measurements were performed separately on the different zones of the root apex: calyptra, RAM and TZ until about 500 μm from the junction calyptra-root apex. Signal intensity measurements were carried out with Leica Application Suite X software (LAS X).

A minimum of 50 seedlings for each sample from three independent replicates was analyzed. The results represent the mean value (± standard deviation) of three independent biological replicates. Asterisks indicate significant pairwise differences using Student’s *t-*test (^∗^*P* < 0.05; ^∗∗^*P* < 0.01; ^∗∗∗^*P* < 0.001).

### RNA Isolation and Reverse Transcription

Primary roots of *A. thaliana* seedlings germinated on control (Ctrl) and on Cd containing medium (25–50 μM respectively) and sampled at 8 DAG were used. Total RNA was isolated from 100 mg of plant tissue and processed with the RNeasy Plant Mini kit (Qiagen, Hilden, Germany) according to the manufacturer’s instructions. RNA was treated with DNase I (Roche, Milan Italy) to remove contaminating genomic DNA. DNAse I was inactivated at 70°C for 15 min. RNA was precipitated and finally resuspended in 40 μL of RNase-free water. RNA quality was checked by agarose gel electrophoresis and quantified by spectrophotometry (NanoDrop technologies). Total RNA (3 μg) from each sample was interacted with SuperScript III Reverse Trascriptase and oligo dT (22) for cDNA synthesis, according to the manufacturer’s instructions (Invitrogen, Milan, Italy).

### Quantitative Real-Time PCR (qRT-PCR)

Quantitative real-time PCR (qRT-PCR) was performed as reported in Bruno et al. (2014) using a STEP ONE instrument (Applied Biosystems). Primers used for qRT-PCR analysis were designed using Primer3 software, according to Yokoyama and Nishitani (2001). Experimentally, optimal primers were identified based upon their ability to meet several standards: (a) robustness: successful amplification over a range of annealing temperatures, (b) specificity: generation of a single significant peak in the melting curve, and (c) consistency: highly reproducible Ct values within the reactions of a triplicate. The average amplification efficiency of each primer pair was determined, and primers performing poorly were replaced. The average efficiency of all the primer pairs discussed in this study ranged between 0.95 and 1.0. After checking independent trials of several housekeeping genes, AT2G28390 (MONENSIN SENSITIVITY1, *SAND*) produced the most reproducible results across various cDNAs, and was used as a normalization control (Remans et al., 2008). The primer sequences are reported in Supplementary Table 1.

Amplification reactions were prepared in a final volume of 20 μL: Power SYBR Green PCR Master Mix (Applied Biosystems) (2X), dATP-dCTP-dGTP (0.4 mM) and dUTP (0.8 mM), each primer (0.4 μM) and cDNA (25 ng). All reactions were run in triplicate in 48-well reaction plates, and negative controls were included. The cycling parameters were as follows: one cycle at 95°C for 3 min to activate the Taq enzyme, followed by 40 cycles of denaturation at 95°C for 10 s and annealing-extension at 60°C for 30 s. After the reaction, in order to confirm the existence of a unique PCR product, the ‘melting curve’ (Lekanne Deprez et al., 2002) was evaluated by an increase of 0.5°C every 10 s, from 60 to 95°C. A unique ‘melting peak’ was obtained in every reaction and the PCR products were verified by 1% agarose gel electrophoresis. The results were analyzed using STEP One Software 2.0 (Applied Biosystems), using the 2^-ΔΔCt^ method (Livak and Schmittgen, 2001). The results represent the mean value (± standard deviation) of three independent biological replicates. Asterisks indicate significant pairwise differences using Student’s *t*-test (^∗^*P* < 0.05; ^∗∗^*P* < 0.01; ^∗∗∗^*P* < 0.001).

## Results

### Root Growth

**FIGURE 1:**
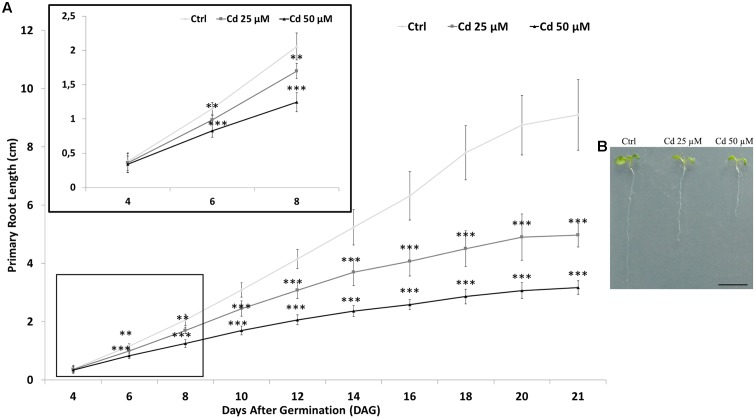
**(A)** Root length of *Arabidopsis thaliana* seedlings germinated on growth medium as control (Ctrl) and on medium added with 25 or 50 μM Cd for 21 days after germination (DAG). In the box, higher magnification of graph from 4 to 8 DAG is showed. **(B)** Picture of Ctrl and Cd-treated seedlings at 8 DAG. The results represent the mean value (± standard deviation) of three independent biological replicates. Asterisks indicate significant pairwise differences using Student’s *t*-test (^∗^*P* < 0.05; ^∗∗^*P* < 0.01; ^∗∗∗^*P* < 0.001). Scale bar **(B)** 0.5 cm.

Root growth was monitored by measuring primary root length up to 21 days after germination (DAG) in *A. thaliana* seedlings germinated: (i) on growth medium as control (Ctrl); ii) on growth medium added with 25 or 50 μM Cd. Starting from the 6th DAG, a significant and dose-dependent reduction of root length was observed in seedlings exposed to Cd compared to the Ctrl (**Figure 1** box). At the end of heavy metal exposure (21 DAG), the reduction in root length was about two and three folds for seedling treated with 25 or 50 μM Cd, respectively (**Figure 1**).

### Root Meristem Size and Pattern

**FIGURE 2:**
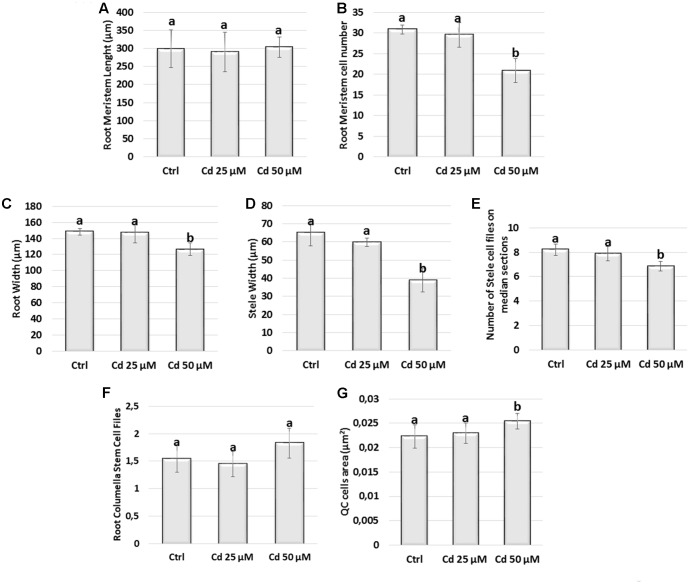
**(A)** Meristem length (μm), **(B)** meristem cell number, **(C)** meristem width (μm), **(D)** stele width (μm), **(E)** stele cell files number, **(F)** calyptrogen layers number, **(G)** quiescent center cell area (μm^2^), in roots of *A. thaliana* seedlings germinated on growth medium as control (Ctrl); and on medium added with 25 or 50 μM Cd for 8 days after germination (DAG). Data present the mean ± standard deviation (SD) of three independent experiments. Statistical analysis was performed by using one-way ANOVA with Tukey *post hoc* test (*P* ≤ 0.05) after Shapiro–Wilk normality test. Means with the same letter are not significantly different at *P* < 0.05.

**FIGURE 3:**
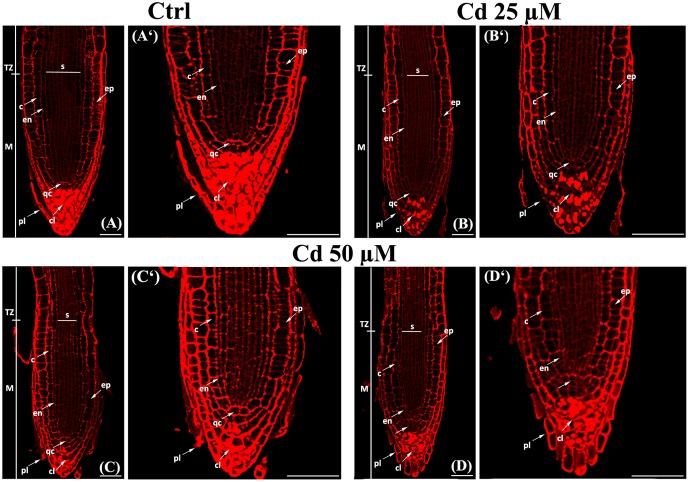
Confocal laser images of primary root tip in *A. thaliana* seedlings germinated **(A)** on growth medium as control (Ctrl) and on medium added with **(B)** 25 and **(C,D)** 50 μM Cd for 8 days after germination. **(A**^‘^**–D**^‘^) Higher magnification of **(A–D)**, respectively. cl, columella; c, cortex; en, endodermis; ep, epidermis; M, meristematic zone; pl, cap peripheral layers; qc, quiescent center; s, stele; TZ, transition zone. Scale bars **(A–D)** 46 μm; **(A**^‘^**–D**^‘^) 53 μm.

Preliminarily, by monitoring when root meristem reached a fixed number of cells, we were able to define that in control and Cd-treated samples root meristem development was fully accomplished at 5/6 DAG and 7/8DAG respectively. Therefore, in order to analyze comparable stages, 8 DAG age was selected to analyze the effects induced by the heavy metal on root meristem size and pattern (**Figures 2**, **3**).

Firstly, we evaluated meristem length by gauging, along the single cortex layer, both the distance and the cell number extending from QC to TZ where cell elongation is starting (**Figures 2**, **3**). We observed that the distance from QC to TZ was quite comparable in treated vs. Ctrl roots (**Figure 2A**). However, at the highest Cd concentration (Cd 50 μM) the number of cortical cells was significantly smaller in Cd-exposed vs. Ctrl roots (**Figure 2B**). In line with this result, meristematic cells were bigger in 50 μM Cd-treated roots than in Ctrl ones (**Figure 3**). Only a slight reduction in the distance from QC to TZ was observed in root meristems exposed to Cd 25 μM although differences were not statistically significant (**Figures 2A,B**).

Concerning the radial pattern, root width was taken into account and evaluated as the cross diameter at the level of TZ (**Figures 2**, **3**). It resulted smaller in roots grown in presence of Cd 50 μM (**Figures 2C**, **3**) mainly due to a reduction in the diameter of stele (**Figure 2D**) which was formed by a lower number of cell files compared to Ctrl roots (**Figure 2E**). No statistically significant differences were detected in roots exposed to Cd 25 μM.

All together these results indicated that at 8 DAG and at the highest concentration (50 μM) Cd impacted on the size and pattern of root meristem while in roots exposed to the lowest concentration (25 μM) similar effects were not yet significant despite a similar trend.

### Aberrant Size and Shape of QC Cells and Surrounding Cells

**FIGURE 4:**
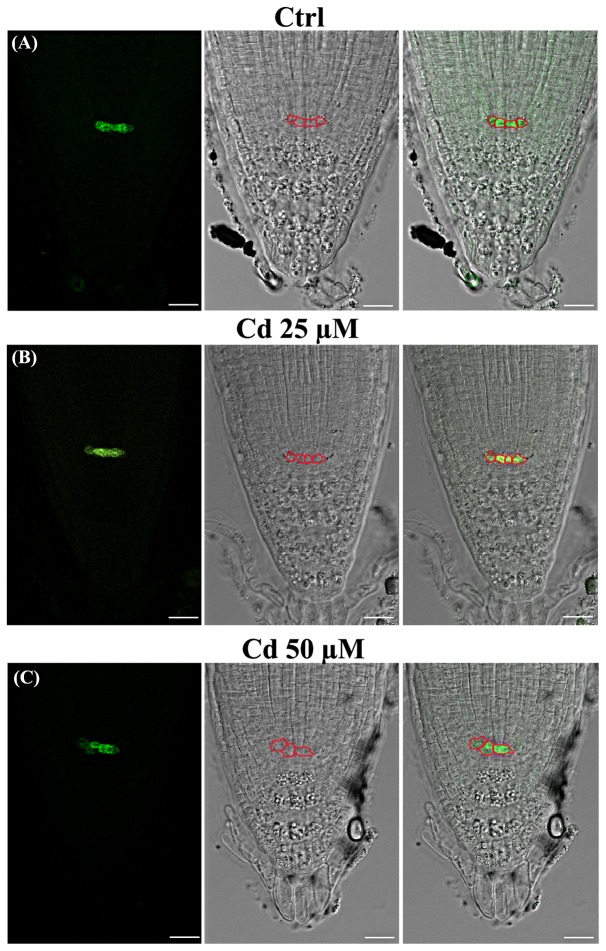
Images of primary root tip in seedlings of *A. thaliana pWOX5::GFP* transgenic line germinated **(A)** on growth medium as control (Ctrl) and on medium added with **(B)** 25 and **(C)** 50 μM Cd for 8 days after germination. From left to right: confocal laser image; transmission image; merged image. Boxed cells expressing GFP signal identify QC cells. Scale bars 23 μm.

Attention was then focused on the QC, which acts as the organizing center of SCN and root meristem (van den Berg et al., 1997; Stahl and Rüdiger, 2010). To this aim, in addition to wild type (**Figure 3**) we used a *pWOX5::GFP* transgenic line of *A. thaliana* expressing a QC specific marker (Blilou et al., 2005), exposed or not to Cd from germination to 8DAG (**Figure 4**).

According to literature (van den Berg et al., 1997), a canonical QC formed by 4 cells was observed in the untreated roots of both wild type (**Figure 3A**^‘^ arrow) and transgenic lines (**Figure 4A**). The number of QC cells did not change in roots exposed to Cd 25 μM (**Figures 3B**^‘^ arrow, **4B**), whereas a reduction to not more than two cells (**Figures 3C**^‘^ arrow, **4C**), coupled to a significant increase of QC cell area (**Figures 2G**, **3C**^‘^), was instead detected in roots exposed to Cd 50 μM. In few cases, QC cells were totally undetectable following Cd 50 μM treatment, thus suggesting a global miss identity of QC (**Figure 3D**^‘^). Globally, these results are consistent with the reduced number of cell files induced by Cd 50 μM in the stele (**Figures 2E**, **3C**), whose initials are directly in contact with QC.

It has also been demonstrated that differentiation fate of the cells surrounding the QC is highly dependent on the same QC (van den Berg et al., 1997; Bitonti et al., 2006). Consistently, in Cd-treated roots a reduction of cap peripheral cell lines was observed together with a delayed differentiation of columella cells, as evidenced by a reduction of statocytes accumulation (**Figure 3**). Moreover, in the root where QC was undetectable, three/four-layers of large cells devoid of statocytes were observable below calyptrogen place (**Figure 3D**^‘^ arrow).

All together, these results suggest that Cd 50 μM toxicity on root growth and both longitudinal and radial pattern is likely dependent on the impact on QC cells.

### Cd Impact on SCARECROW Expression Pattern

SCARECROW (SCR) is a transcription factor of the GRAS family which is involved in the specification of QC identity, and hence in stem cell niche and meristem maintenance as well as in the definition of root radial patterning (Di Laurenzio et al., 1996; Sabatini et al., 2003). Therefore, based on the above results, we planned to investigate the effects of Cd on the SCR expression pattern by using *pSCR::SCR-GFP* transgenic line exposed or not to Cd from germination to 8 DAG.

**FIGURE 5:**
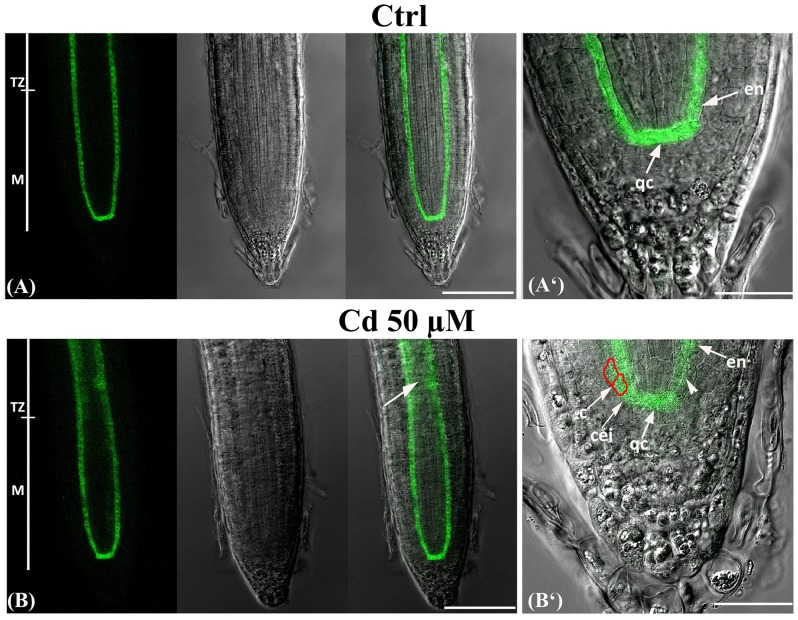
Expression of SCARECROW in the primary root of seedlings of *A. thaliana pSCR::SCR-GFP* transgenic line germinated **(A)** on growth medium as control (Ctrl) and on medium added with **(B)** 50 μM Cd for 8 days after germination. From left to right: confocal laser image; transmission image; merged image; higher magnification. c, cortex; cei, cortex and endodermis initial; en, endodermis; M, meristematic zone; qc, quiescent center; TZ, transition zone. Scale bars **(A,B)** 110 μm; **(A**^‘^**,B**^‘^) 42 μm.

In Ctrl roots, GFP signal resulted to be typically confined to the QC cells, the cortex/endodermis initials and along the endodermis (Di Laurenzio et al., 1996; Pysh et al., 1999) (**Figures 5A,A**^‘^). No differences were detected under Cd 25 μM treatment (data not shown). By contrast, in 50 μM Cd-treated roots, a mislocalisation of GFP signal was observed since it was ectopically expressed in the stele at the level of TZ (**Figure 5B** arrow) detected also in committed cortex cells (**Figure 5B**^‘^ boxed cells), while appeared very low in some endodermis cells proximal to QC (**Figure 5B**^‘^ arrow head).

### Cd Impact on Auxin Distribution

**FIGURE 6:**
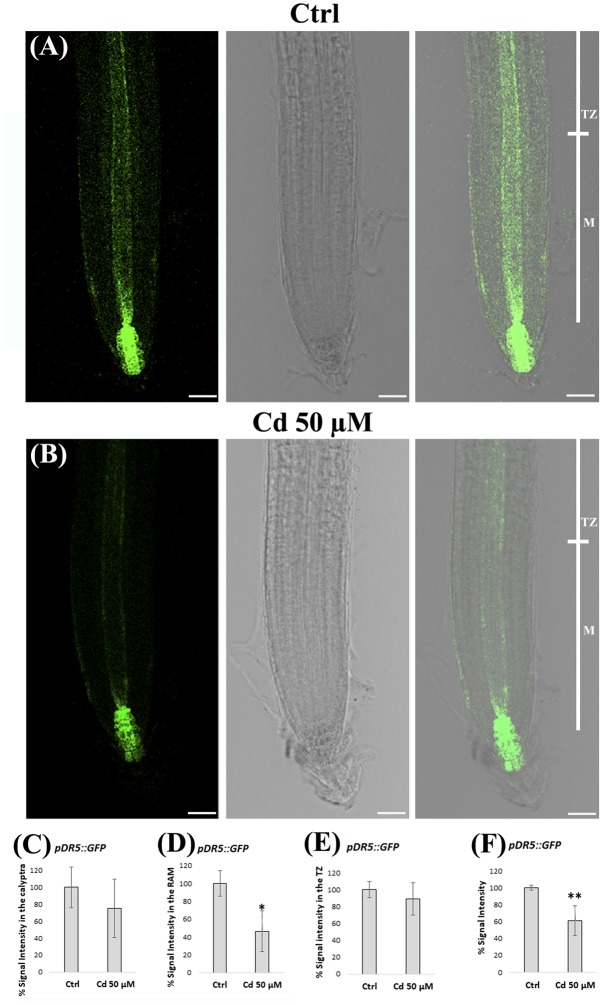
**(A,B)** Images of primary root apex in seedlings of *A. thaliana pDR5::GFP* transgenic line germinated **(A)** on growth medium as control (Ctrl) and on medium added with **(B)** 50 μM Cd for 8 days after germination. From left to right: confocal laser image; transmission image; merged image. *pDR5::GFP* relative signal intensities (normalized to Ctrl), in **(C)** the calyptra, **(D)** the RAM, **(E)** the TZ and **(F)** the whole root apex, of above mentioned Ctrl and Cd-treated roots. The results represent the mean value (± standard deviation) of three independent biological replicates. Asterisks indicate significant pairwise differences using Student’s *t-*test (^∗^*P* < 0.05; ^∗∗^*P* < 0.01; ^∗∗∗^*P* < 0.001). Scale bars 46 μm.

Based on the pivotal role of auxin in root morphogenesis, we planned to investigate whether the alterations in root patterning and QC identity, detected at 8 DAG in roots exposed to Cd 50 μM, could be related to an affected auxin signaling and distribution. To this aim, we used *A. thaliana* line expressing the *pDR5::GFP* auxin inducible reporter exposed or not to heavy metal treatment (**Figures 6A,B**). To estimate quantitative spatial differences, signal intensity was measured at the level of calyptra, RAM and TZ, separately (**Figures 6C–E**). We observed that *pDR5::GFP* signal was significantly reduced in the root apex of Cd-treated vs. Ctrl roots (**Figures 6A–F**), mainly in the RAM (**Figures 6A,B,D**) and more specifically along the stele and at SCN level (**Figures 6A,B**). This signal distribution pattern clearly indicated that the establishment of auxin gradient in the root apex was impaired following heavy metal exposure.

**FIGURE 7:**
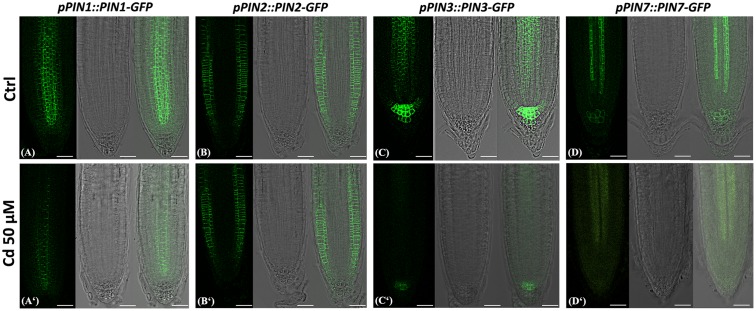
Images of primary root tip in seedlings of *A. thaliana pPIN1::PIN1-GFP, pPIN2::PIN2-GFP, pPIN3::PIN3-GFP, pPIN7::PIN7-GFP* transgenic lines germinated **(A–D)** on growth medium as control (Ctrl) and **(A**^‘^**–D**^‘^) on medium added with 50 μM Cd for 8 DAG. From left to right: confocal laser image; transmission image; merged image. Scale bars 46 μm.

**FIGURE 8:**
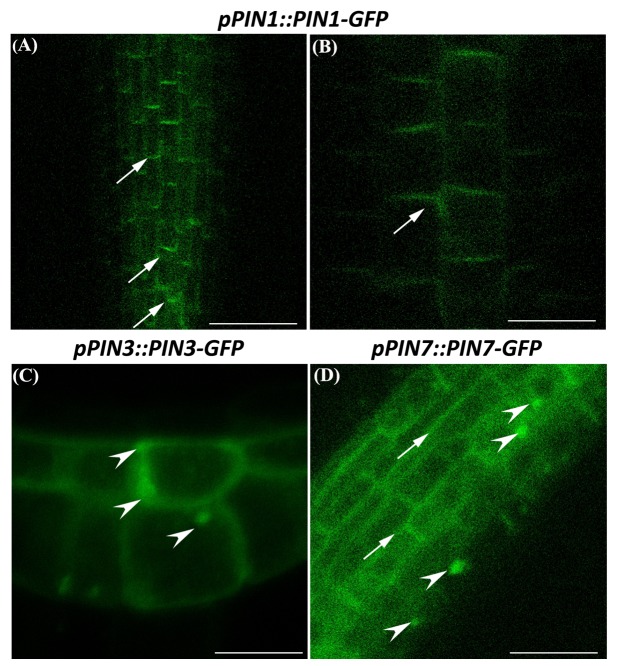
Subcellular localization of PIN1, **(A,B)**, PIN3 **(C)** and PIN7 **(D)** proteins in primary root of *A. thaliana* seedlings germinated on growth medium as control (Ctrl); and on medium added with Cd 50 μM for 8 DAG. Arrow on lateral localization of PIN; arrow head on PINs intracellular accumulation. Scale bars **(A)** 12 μm; **(B–D)** 25 μm.

This observation prompted us to investigate the distribution of PIN-FORMED (PIN) proteins, the main polar auxin efflux carriers (Benková et al., 2003; Petrásek and Friml, 2009) through the use of the following *A. thaliana* transgenic reporter lines: *pPIN1::PIN1-GFP, pPIN2::PIN2-GFP, pPIN3::PIN3-GFP, pPIN7::PIN7-GFP*. The analysis by confocal microscopy showed that globally PINs presence and distribution clearly differed in Cd-treated vs. Ctrl roots (**Figure 7**). In particular, PIN1 canonically resided at the basal end of provascular cells extending until the SCN (Friml et al., 2002a), but signal intensity was greatly reduced in Cd-treated roots compared to the Ctrl (**Figures 7A,A**^‘^). No significant differences were instead detected for PIN2, whose signal was typically localized at the apical side of epidermal and lateral root cap cells and predominantly basally in the cortical cells in both treated and untreated roots (**Figures 7B,B**^‘^). Severe changes were observed for PIN3 signal, which in Ctrl roots was detectable in SCN, in two/three cell tiers of the columella cells and at the basal side of vascular cells as previously reported (Friml et al., 2002b), whereas in Cd-treated roots signal was almost absent in the stele and weakly confined to one/two columella cell tiers (**Figures 7C,C**^‘^). As for PIN7 expression, an intense signal was typically detected in columella and in provascular cells of Ctrl roots while it appeared weakly spread in the stele and almost absent in the columella of Cd-treated roots (**Figures 7D,D**^‘^). In addition, a weak lateral localization of PIN1, PIN3 and PIN7 (**Figure 8** arrows) and, occasionally, an intracellular accumulation of PIN3 and PIN7 transporters (**Figure 8** arrow heads) were also observed in treated roots.

**FIGURE 9:**
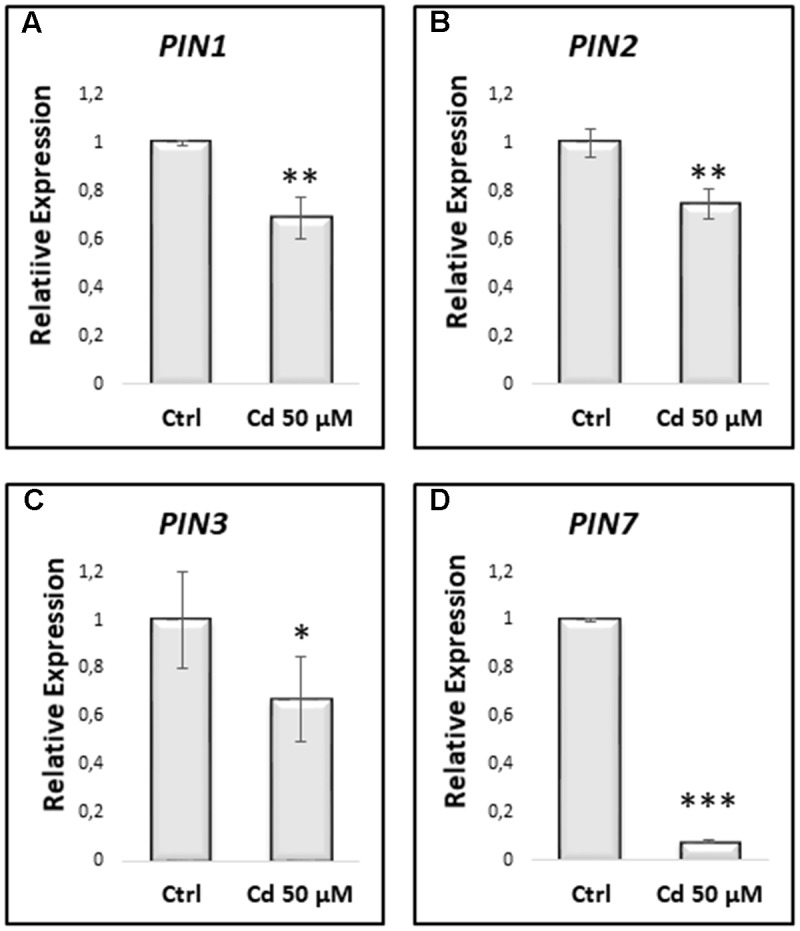
Relative expression by qRT-PCR of *PIN1* **(A)**, *PIN2* **(B)**, *PIN3* **(C)** and *PIN7* **(D)**, in primary roots of *A. thaliana* seedlings germinated on growth medium as control (Ctrl); and on medium added with 50 μM Cd for 8 DAG. Mean expression levels were calculated from three biological replicates, obtained from three independent experiments. Results were analyzed using STEP One Software 2.0 (Applied Biosystems), using the 2^-ΔΔCt^ method (Livak and Schmittgen, 2001). The results represent the mean value (± standard deviation) of three independent biological replicates. Asterisks indicate significant pairwise differences using Student’s *t*-test (^∗^*P* < 0.05; ^∗∗^*P* < 0.01; ^∗∗∗^*P* < 0.001).

In line with the downregulation of PIN proteins expression, also the genes encoding these proteins were found to be downregulated in Cd-treated vs. Ctrl roots (**Figure 9**).

### Cd Impact on Cytokinin Signaling

**FIGURE 10:**
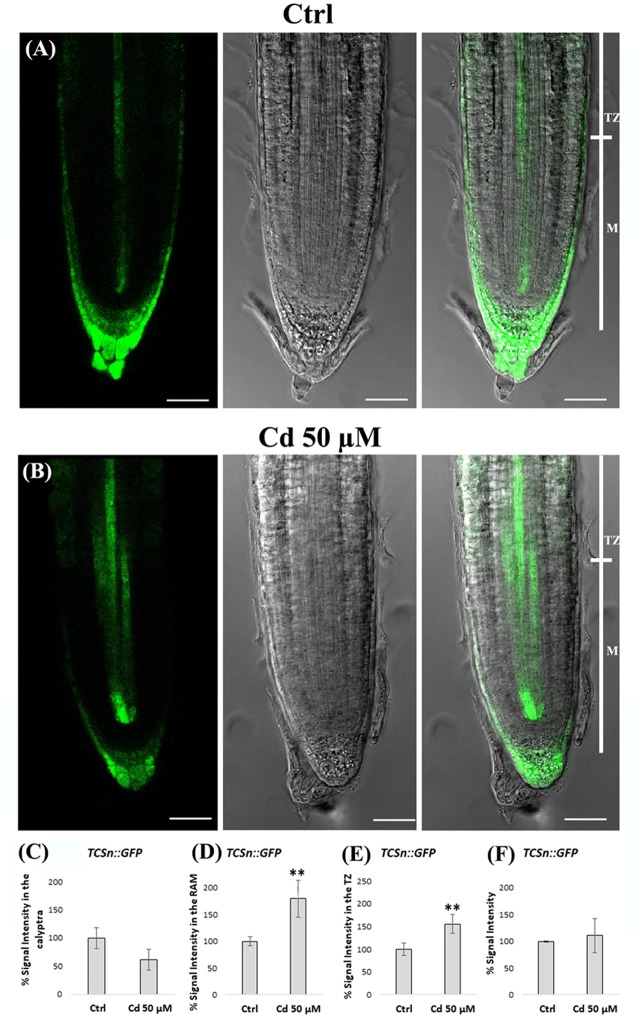
Expression of *TCSn* cytokinin synthetic sensor in the primary root apex of seedlings of *A. thaliana TCSn::GFP* transgenic line germinated **(A)** on growth medium as control (Ctrl) and **(B)** on medium added with 50 μM Cd for 8 days after germination (DAG). From left to right: confocal laser image; transmission image; merged image. Scale bars 46 μm. *TCSn::GFP* relative signal intensities (normalized to Ctlr), in **(C)** the calyptra, **(D)** the RAM, and **(E)** the TZ, and **(F)** the whole root apex of above mentioned Ctrl and Cd-treataed roots. The results represent the mean value (± standard deviation) of three independent biological replicates. Asterisks indicate significant pairwise differences using Student’s *t*-test (^∗^*P* < 0.05; ^∗∗^*P* < 0.01; ^∗∗∗^*P* < 0.001).

Finally, due to the antagonistic effect of auxin and cytokinin on root growth and morphogenesis we analyzed some aspects of cytokinin signaling pathway by using a *TCSn::GFP* transgenic line of *A. thaliana* carrying a specific synthetic sensor. Seedling roots exposed or not to Cd from germination to 8 DAG were used and GFP signal intensity was measured at the level of calyptra, RAM and TZ, separately (**Figures 10A–F**). A quite comparable level of signal was observed when considering the whole root apex. However, and more interestingly, clear differences were evident when considering the different root zones. Namely, in Cd-treated roots vs. Ctrl roots signal intensity resulted lower in the calyptra but significantly higher in both RAM and above all in TZ. Moreover, at histological level, signal resulted more largely extended, mainly in the stele and SCN (**Figures 10A,B**). This signal distribution pattern clearly indicated that cytokinin signaling in the root apex was strongly altered by Cd treatment.

## Discussion

Cadmium is one of the most widespread pollutant in both terrestrial and marine environment, and its inhibitory effect on plant root growth has been widely assessed (Yuan and Huang, 2016). It is known that root growth is dependent on RAM maintenance, which is assured by a balance between the production of new meristematic cells and their displacement toward the differentiation (Bitonti and Chiappetta, 2011; Moubayidin et al., 2016). This balance is not solely the result of a linear hormone pathway, but rather the output of multiple pathways, in which auxin and cytokinins play a prominent role (Benjamins and Scheres, 2008; Dello Ioio et al., 2008; Benková and Hejátko, 2009). Accordingly, in the present work, we demonstrated that Cd-induced inhibition of root growth is related to an altered homeostasis of auxin/cytokinin signaling, which in turn impacts on meristem size and stem cell niche activity. Namely, through the use of *pDR5::GFP* auxin responsive reporter we demonstrated that the establishment of auxin maximum at the root apex was impaired in roots exposed to Cd. This result is in line with previous data in literature showing that in *A. thaliana* roots a short Cd exposure (12 h) at comparable concentrations caused a reduction of RAM size associated to a reduced auxin level in the root tip (Besson-Bard et al., 2009; Hu et al., 2013; Yuan and Huang, 2016). By contrast, at lower Cd concentration (10 μM), an increased auxin level was detected in the whole root of treated seedlings where an enhanced lateral root production was also observed (Sofo et al., 2013), likely as compensatory mechanism to the reduced growth of primary root. Although obtained under different treatment conditions, altogether these results strongly suggest that alteration of auxin distribution more than its total level (amount) is the most relevant effect of Cd treatment. Accordingly, in the apex of Cd-treated roots we detected a downregulation at both transcriptional and post-transcriptional level of PIN proteins which are central rate-limiting components of auxin transport (Blilou et al., 2005; Grieneisen et al., 2007; Petrásek and Friml, 2009). That is different from literature data showing an effect of Cd on PINs expression only at post-transcriptional level when applied at a lower concentration and short time compared to the present study (Yuan and Huang, 2016).

Concomitantly and very interestingly, under Cd exposure we observed an enhancement of cytokinin signaling in the root apex, which is consistent with the overproduction of such hormones detected in both root and shoot of Cd- treated plants of *A. thaliana* by Sofo et al. (2013). Conceivably, the enhanced cytokinin signaling could account for PINs transcriptional downregulation that we observed, given that it promotes SHY2 expression, which negatively regulates PIN 1, 3,7 genes (Benjamins and Scheres, 2008; Dello Ioio et al., 2008). On this basis, we propose that the alterations detected in the RAM of treated roots relied on Cd capacity to affect auxin maximum establishment by enhancing cytokinin signaling which in turn inhibits PIN carriers expression and auxin transport to the root apex.

In addition, for the first time a somewhat loss of polarity and intracellular accumulation of PIN proteins was also observed in Cd-treated roots. It has been long established that plasma membrane-resident PIN proteins are targeted to their final destinations through complex endomembrane trafficking mechanisms (Kleine-Vehn and Friml, 2008; Luschnig and Vert, 2014). Moreover, they are frequently dynamically endocytosed and either recycled or transcytosed or sorted through late endosome to a vacuolar-targeted pathway for degradation (Kleine-Vehn and Friml, 2008; Kleine-Vehn et al., 2008a,b. In this context, it is worthy to recall that actin filaments have been found to play an important role in recycling of PIN1 and PIN3 proteins as well as in the PIN1 intracellular movement in *A. thaliana* roots (Geldner et al., 2001; Friml et al., 2002a; Kleine-Vehn et al., 2006). Very interestingly, there are evidences that Cd strongly impact on cytoskeleton (Fan et al., 2011; Wan and Zhang, 2012). Therefore, the detected alteration in PINs polarity and distribution could be somehow related also to the effect of Cd on cytoskeleton and consequently on PIN trafficking, thus contributing to affect auxin transport to the root apex.

Accordingly to the described alteration in auxin/cytokinin interplay, RAM cell number along the cortex was lower in the roots exposed to the heavy metal compared with control ones. Moreover, these cells were bigger in treated than in control roots, suggesting that they are losing the meristematic features as verified also in the ground meristem of *A. thaliana* lateral roots, even applying a lower Cd concentration (Zanella et al., 2016). Therefore, the increase in cell area could represents a kind of compensatory mechanism to a reduced potential for proliferation.

Moreover, our results also show that under the treatment that we applied starting from germination, clear effects on stem cell niche were induced by the highest Cd concentration (50 μM) at the 8 DAG. In this context, it must be highlighted that root patterning is tightly related to the activity of stem cell niche formed by initials which divide continuously, producing at each division one cell that continues to act as an initial. The number of RAM initials varies according to species (Webster and MacLeod, 1980), but they are almost permanent in position and include the QC which represents the generating center of root pattern and architecture. In *A. thaliana* root QC is formed by four cells (van den Berg et al., 1997). The use of GFP reporter lines expressing a QC specific marker allowed us to verify that following Cd 50 μM treatment QC was formed by a lowered number of cells with an altered size and shape. Moreover, some time QC appears almost depleted. All together these results suggest that a miss identity of stem cells is underway. According to such hypothesis, alterations in root pattern have been observed in Cd-treated roots in both proximal and distal direction. Indeed, a delayed differentiation of columella cells sometime associated to three/four-layered calyptrogen formed by large isodiametric cells was observed in Cd-treated roots, consistently with the role of QC in delaying differentiation of surrounding cells (van den Berg et al., 1997; Bitonti et al., 2006). On the other side, we observed that Cd exposure caused a reduction of cell files in the stele, likely related to the reduction of QC cell number, thus affecting also the radial pattern of the root.

In this context, it appears of interest the deregulation of SCR expression observed in Cd-treated roots. It is known that SCR is involved in formative stem cell division and exerts a fine control on ARR1 levels in the roots (Heidstra et al., 2004; Cui et al., 2007; Cruz-Ramírez et al., 2012). Namely, it directly suppresses ARR1 in the QC maintaining stem cell niche, while at the TZ activates ARR1 transcription by modulating non-autonomously the ARR1 transcript levels via auxin and by sustaining gibberellin signals from the endodermis (Moubayidin et al., 2013, 2016). Interestingly, in line with this picture, in Cd-treated roots SCR signal was somehow weak in some cell of the stem niche, which resulted reduced, whereas was ectopically expressed in the stele where cytokinin signal was enhanced.

## Conclusion

The present work provides evidence that Cd toxicity on root growth is dose and time dependent and strongly depends on the effects induced on RAM stem cell niche. At the molecular level for the first time Cd-induced effects has been related to a misexpression of SCR transcription factors, as well as to an altered homeostasis of auxin/cytokinin signaling which both play a relevant role in the regulatory circuit underlying RAM maintenance and activity (Di Laurenzio et al., 1996; Drisch and Stahl, 2015; Moubayidin et al., 2016). Notably, Cd resulted to modulate SCR expression in different ways from different root tissues (proximal vs. distal), thus supporting the idea that cell/tissue-specific co-factor and/or epigenetic mechanisms could be involved (Cruz-Ramírez et al., 2012; Greco et al., 2012). Therefore, much further work will be required to fully dissect the molecular framework of Cd effects on root growth and pattern.

## Author Contributions

LB, MG, and MB designed research; LB, MP, IF, LL, and MG performed research; LB, MP, AC, and MB analyzed data and discussed results; LB, MP, and MB wrote the paper. All authors contributed to improving the paper and approved the final manuscript.

## Conflict of Interest Statement

The authors declare that the research was conducted in the absence of any commercial or financial relationships that could be construed as a potential conflict of interest.

## Footnotes

1 https://imagej.net
