# Supplementary material for: In Arabidopsis thaliana Cadmium Impact on the Growth of Primary Root by Altering SCR Expression and Auxin-Cytokinin Cross-Talk
Source: Front Plant Sci. 2017 Jul 27;8:1323. doi: 10.3389/fpls.2017.01323 (PMC5529362; doi:10.3389/fpls.2017.01323)
Supplement: Supplementary file 1 [file Table_1.DOCX]

**Supplementary Table 1** List of Primers used for qPCR

| **Name** | **Primer** | **Sequence** |
| --- | --- | --- |
| PIN1 | FW | 5’-TACTCCGAGACCTTCCAACTACG-3’ |
| PIN1 | RW | 5’-TCCACCGCCACCACTTCC-3’ |
| PIN2 | FW | 5’-TTTCTCCACGCAAATTCTTTG-3’ |
| PIN2 | RW | 5’-GCTGCTCTTCCTCAAGGAATC-3’ |
| PIN3 | FW | 5’GAGGGAGAAGGAAGAAAGGGAAAC-3 |
| PIN3 | RW | 5’- CTTGGCTTGTAATGTTGGCATCAG-3’ |
| PIN4 | FW | 5’-GATGCTGGTCTTGGAATGG-3’ |
| PIN4 | RW | 5’-CCTGAACGATGGCTATACG-3’ |
| PIN7 | FW | 5’-CTTGGTATGGCAATGTTCAG-3’ |
| PIN7 | RW | 5’-CACACGCAATAGGTCTC-3’ |
| SAND | FW | 5’- AACTCTATGCAGCATTTGATCCACT -3’ |
| SAND | RW | 5’- TGATTGCATATCTTTATCGCCATC -3’ |
